# Visual information processing of 2D, virtual 3D and real‐world objects marked by theta band responses: Visuospatial processing and cognitive load as a function of modality

**DOI:** 10.1111/ejn.16634

**Published:** 2024-12-09

**Authors:** Joanna Kisker, Marike Johnsdorf, Merle Sagehorn, Thomas Hofmann, Thomas Gruber, Benjamin Schöne

**Affiliations:** ^1^ Experimental Psychology I, Institute of Psychology Osnabrück University Osnabrück Germany; ^2^ Industrial Design, Engineering and Computer Science University of Applied Sciences Osnabrück Osnabrück Germany; ^3^ Department of Psychology Norwegian University of Science and Technology Trondheim Norway

**Keywords:** 2D, neural oscillations, reality, virtual reality, visuospatial processing

## Abstract

While pictures share global similarities with the real‐world objects they depict, the latter have unique characteristics going beyond 2D representations. Due to its three‐dimensional presentation mode, Virtual Reality (VR) is increasingly used to further approach real‐world visual processing, yet it remains unresolved to what extent VR yields process comparable to real‐world processes. Consequently, our study examined visuospatial processing by a triangular comparison of 2D objects, virtual 3D objects and real 3D objects. The theta band response (TBR) was analysed as an electrophysiological correlate of visual processing, allowing for the differentiation of predominantly stimulus‐driven processes mirrored in the evoked response and internal, complex processing reflected in the induced response. Our results indicate that the differences between conditions driven by sensory features go beyond a binary division into 2D and 3D materials but are based on further sensory features: The evoked posterior TBR differentiated between all conditions but revealed fewer differences between processing of real‐world and VR objects. Moreover, the induced midfrontal TBR indicated higher cognitive load for 2D objects compared to VR and real‐world objects, while no difference between both latter conditions was revealed. In conclusion, our results demonstrate that the transferability of 2D‐ and VR‐based findings to real‐world processes depends to some degree on whether predominantly sensory stimulus features or higher cognitive processes are examined. Yet although VR and real‐world processes are not to be equated based on our results, their comparison yielded fewer significant differences relative to the PC condition, advising the use of VR to examine visuospatial processing.

## Graphical abstract

Our study examines modality‐specific differences in visual information processing by comparing electrophysiological responses to 2D objects, virtual 3D objects and real‐world objects. Our results indicate that the differences between conditions driven by sensory features go beyond a binary division into 2D and 3D materials. Moreover, they indicate higher cognitive load for 2D objects compared to VR and real‐world objects, while both of the latter did not significantly differ.

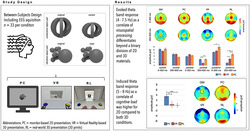

**Abbreviations**
BF: bayes factor
EEG: electroencephalography
EOG: electrooculogram
eTBR: evoked theta band response
HMD: head‐mounted display
iTBR: induced theta band response
ITI: inter‐trial‐interval
PC: personal computer
RL: real‐life
TBR: theta band response
TF: time‐frequency
VR: virtual reality

## 1 INTRODUCTION

Previous research on the visual perception and processing of objects and thus, on the respective neural mechanisms is predominantly based on two‐dimensional objects, i.e., planar pictures of certain objects and their properties, while everyday percepts result from a three‐dimensional environment (Nastase et al., 2020; Shamay‐Tsoory & Mendelsohn, 2019; Snow & Culham, 2021). Yet besides evidence for global similarities between the visual processing of 2D and 3D objects, recent advancements increasingly acknowledge that real‐world objects have unique features, and consequently effects on visual processing that go beyond what can be revealed using planar pictures as experimental cues (Marini et al., 2019; see also Nastase et al., 2020; Shamay‐Tsoory & Mendelsohn, 2019; Snow & Culham, 2021). In particular, the stereoscopy missing in 2D settings is increasingly addressed by using Virtual Reality (VR) to include realistic visual cues (e.g., Kisker et al., 2024; Peeters, 2018; Schöne et al., 2021; Tromp et al., 2020) while circumventing the affordances of presenting real‐world objects in a controlled manner (see e.g., Marini et al., 2019).

These investigations of whether visual information from conventional 2D and virtual 3D stimuli, i.e., objects presented three‐dimensionally in VR, are distinctly processed focus on oscillatory brain activity in diverse frequency bands as markers of both, processes evoked primarily by the sensory features during stimulus presentation (e.g., Tang et al., 2022), as well as top‐down processes related to complex cognitive processing of the perceived stimuli (e.g., Dan & Reiner, 2017; Malik et al., 2015). Of particular interest is the theta band response (TBR; 4‐8 Hz; e.g., Nyhus & Curran, 2010) as a marker of early perceptual processing in terms of the objects' visuospatial representations (Tang et al., 2022), as well as attention and resource allocation (Bossi et al., 2020; Li et al., 2020), and memory‐related processes like working memory, early stages of encoding (Grunwald et al., 2001; Malik et al., 2015), and cognitive load (Dan & Reiner, 2017; Slobounov et al., 2015).

The majority of studies differentiating the neural correlates of 2D and virtual 3D materials reports a relatively higher TBR to VR‐based than to PC‐based stimulus presentation during different experimental setups and tasks, for example, during passive viewing of either VR or 2D stimuli (Malik et al., 2015;Tang et al., 2022; Xu & Sui, 2021). This difference in the TBR was attributed to spatial information processing and the visual discrimination of 2D and 3D materials (Tang et al., 2022; Xu & Sui, 2021), as well as increased attention and working memory involvement regarding 3D materials (Malik et al., 2015). Similarly, studies including an active task like route learning (Slobounov et al., 2015) or perceptual discrimination of stimuli (Li et al., 2020) report a relatively higher midfrontal TBR during VR conditions compared to PC conditions as well, attributing it to the allocation of more resources under VR conditions facilitating either cognitive control (Slobounov et al., 2015), or attentional processing (Li et al., 2020). In these cases, the relatively higher TBR under VR conditions is discussed as mirroring beneficial effects of using VR for the processing and encoding of the presented stimuli (e.g., Li et al., 2020; Slobounov et al., 2015). However, other findings support the notion that cognitive resource allocation and particularly cognitive load might be lower for 3D materials compared to 2D materials: In contrast to the aforementioned studies, (Dan & Reiner, 2017) report a relatively higher TBR during learning from a 2D video compared to a 3D video, indicating lower cognitive load in the VR condition which is interpreted as being a beneficial effect of using VR for learning as well.

Yet the current knowledge base comes with some limitations. Most importantly, the aforementioned studies seem to consider the entirety of the respective frequency power and hence, do not differentiate between the evoked theta band response (**e**TBR) and the induced theta band response (**i**TBR). The evoked response is phase‐locked to the stimulus onset and thus, predominantly reflects early stages of visual processing. In contrast, induced responses occur with a jitter in latency after stimulus onset, are thus non‐phase‐locked to the stimulus onset and reflect the internal, complex processing of the stimulus (Eckhorn et al., 1990). Both types of oscillations are complementary (Eckhorn et al., 1990). Yet the separate consideration of evoked and induced responses would allow for a concise differentiation of the proposedly associated processes by means of predominantly stimulus‐driven processes like visuospatial processing and higher cognitive processes related to cognitive load and mnemonic processing.

Moreover, previous studies use heterogeneous technological setups for both 2D and VR presentations. The majority utilize conventional desktop presentation for 2D conditions, while (Xu & Sui, 2021) presented the stimulus material two‐dimensionally in VR using a head‐mounted display (HMD). The technological variance is much higher when it comes to VR technologies, with some studies using HMDs (Li et al., 2020; Tang et al., 2022; Xu & Sui, 2021), others using 3D desktops or TVs affording compatible stereo glasses (Dan & Reiner, 2017; Malik et al., 2015; Slobounov et al., 2015) and only few even realizing a real‐life condition for 3D representations (e.g., Marini et al., 2019; see also Schöne et al., 2023). Consequently, it remains to be explored whether the presented results transfer between the diverse approaches or depend to some degree on the mode of 3D presentation. In the latter case, they would primarily allow for the statement that visual information processing differs between 2D material and diverse 3D materials in VR at the electrophysiological level but might not necessarily reveal a consistent and overarching pattern of the neural correlates of 3D object processing. As a consequence, the question arises of whether the findings based on virtual 3D objects presented in VR actually transfer more accurately to real‐world objects than 2D‐based findings.

Accordingly, the study at hand aims to unravel the differences found between visuospatial processing of 2D and 3D objects, expanding previous studies by including a triangular comparison of 2D objects, virtual 3D objects and real‐world 3D objects. To this end, we analyse data from a large‐scale project study during which participants were presented with abstract objects either on a conventional 2D PC screen, in an immersive VR environment or in a real‐world setting realized by means of 3D prints of the stimuli. While the majority of previous studies do not differentiate between induced and evoked oscillatory responses, the study at hand aims to disentangle to what degree the aforementioned differences are driven by the response to sensory stimulus features by means of the eTBR, and to what extent by more complex cognitive processes reflected in the iTBR. Specifically, as a marker of visuospatial representation, we hypothesize that the eTBR will differentiate between 2D and 3D stimuli, reflected in a relatively higher eTBR to virtual 3D and real‐world 3D objects compared to 2D objects (Tang et al., 2022). Based on the findings by Tang et al. (2022), we assume this difference to be most pronounced directly after stimulus onset measured at parietal and posterior sensors. Beyond these phase‐locked processes, the iTBR measured at midfrontal sensors mirrors cognitive load during higher processing of the presented objects, with a relatively higher iTBR reflecting a relatively higher cognitive load (e.g., Dan & Reiner, 2017). However, since previous studies are inconsistent in whether the iTBR is higher to 2D or to virtual 3D objects (e.g., Dan & Reiner, 2017; Slobounov et al., 2015), we aim to shed light on the circumstances under which the cognitive load is higher either under 3D or 2D conditions. Consequently, we propose that the midfrontal iTBR will differentiate between 2D objects and real‐world 3D objects, with the response to virtual 3D objects being more comparable to real‐world objects than to 2D objects.

## 2 METHODS

The current results originate from a large‐scale dataset using the same experimental procedure and data acquisition (for preregistration see: https://osf.io/e64cd/?view_only=cba2d368d21f483a85dc701c0b11d216). The analyses at hand are restricted to a subset of this dataset relevant to the present research question. Companion publications will address other subsets of this comprehensive dataset. Since all publications are based on the same experiment, we aimed for the highest congruence of the in‐depth methodological description, resulting in overlaps between the publications, concerning e.g., figure details depicting the experimental procedure and the methodological details.

### 2.1 A priori power analysis

An a‐priori power analysis was conducted using G*Power (Faul et al., 2007). The expected effect size for the present experiment was estimated at *η* ^*2*^ = .14, hence at the lower bound of a large effect (Cohen, 1988). This estimation was based on previous studies which reported large effects on the electrophysiological correlates on perceptual and mnemonic processes under different modalities (Johnsdorf et al., 2023; Schöne et al., 2023). The estimated sample size had to be suitable for a variety of planned analyses focusing on different aspects of the experiment. Consequently, the power analysis was based on an ANOVA as statistical analysis, an α error probability of 0.05 and a power of 0.95. The analysis revealed a required total sample size of 98 participants. To achieve an equal number of data sets per condition, we aimed for 33 participants per condition.

### 2.2 Participants

One‐hundred and seven participants were recruited via the psychology student e‐mail list, the University's online bulletin board and by students of the bachelor's and master's degree programs of psychology. All participants were screened by the investigators for psychological, psychiatric or neurological disorders and substance intake before participation by means of a short interview and self‐report (anamnesis). Three participants were excluded from participation due to unfulfilled inclusion criteria, one participant prematurely terminated the experiment and four participants had to be excluded due to technical issues. Therefore, the data of *n* = 99 participants (31 male, 68 female) were included in the data analyses (PC: *n* = 33 (60.6% female), *M*_Age_ = 22.48, *SD*_Age_ = 3.06; VR: *n* = 33 (69.7% female), *M*_Age_ = 22.21, *SD*_Age_ = 3.80; RL: *n* = 33 (75.8% female), *M*_Age_ = 22.76, *SD*_Age_ = 2.82). Participants received partial course credits or 20€, and the chance to win one of two 50€ vouchers. The study was conducted in accordance with the declaration of Helsinki and was approved by the local ethics committee (reference: Ethik 5/2023). All participants gave informed written consent before participation.

### 2.3 Stimulus material

**FIGURE 1:**
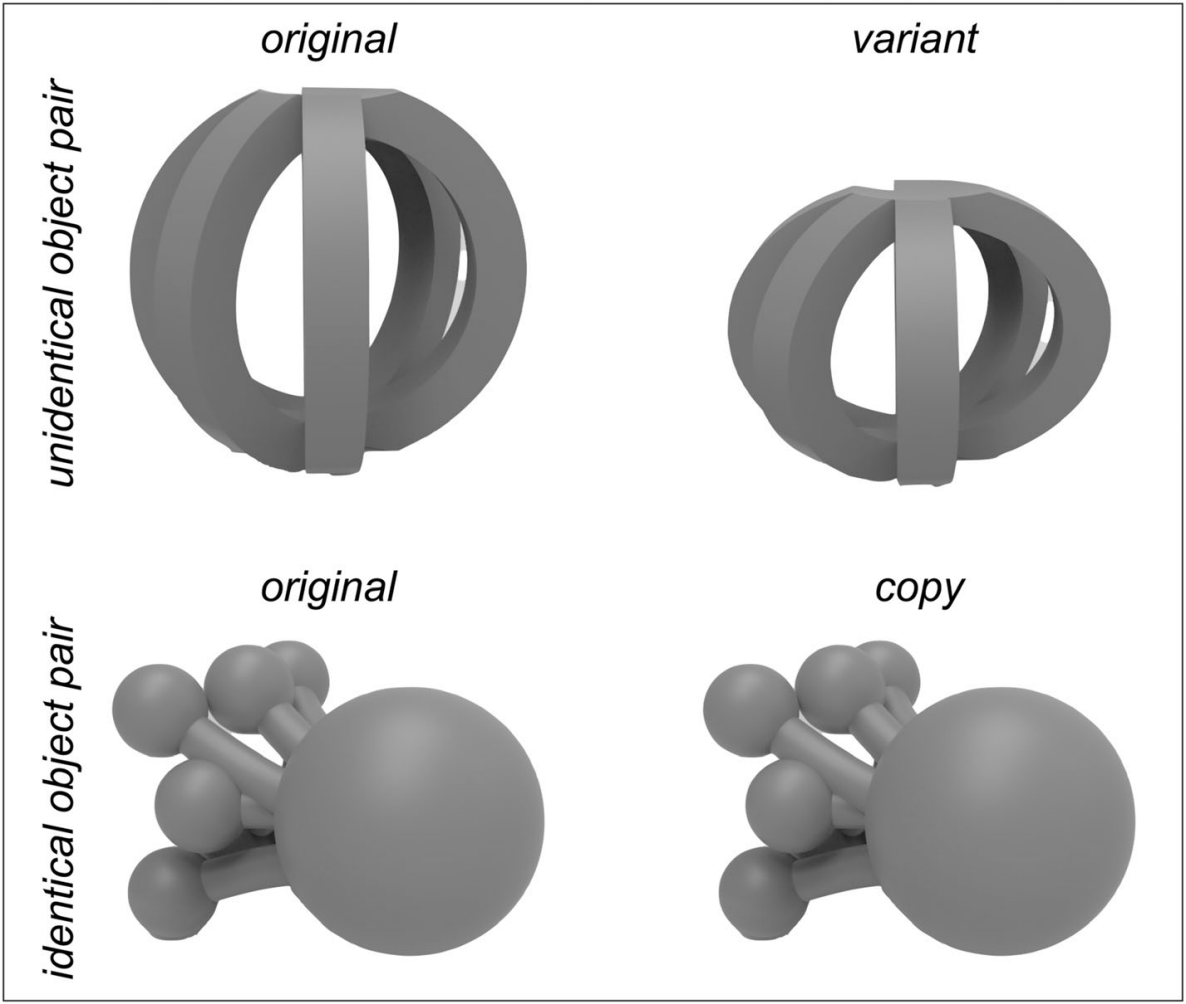
*Exemplary stimuli.* The study material included 40 unidentical object pairs, consisting of an original object and a variant of the original (upper pair; the difference is visible in the height of the variant) and 40 identical pairs consisting of an original object and its copy (lower pair).

The stimuli were designed to be displayed in real‐life (RL), via a PC monitor (PC) and via a VR‐HMD (VR). A set of 160 abstract 3D objects was modelled using Supershapes (version 0.0.3, https://andrewmarsh.com/software/supershapes-web/) and Rhino (version 5; Robert McNeel & Associates, Seattle, WA, USA). All objects were counterchecked for potential semantic associations by two investigators and were excluded and replaced with new objects if at least one investigator associated the abstract object with any non‐abstract object (e.g., an apple, a spinning top, etc.). Half of the objects were used during the encoding session (see below). Of these 80 objects, 40 objects were re‐modelled with a slight variation, offering two versions of the similar object (original and variant, i.e., unidentical object pairs; see Figure 1), while for the further 40 pairs a copy of the original was created (original and copy, i.e., identical object pairs; see Figure 1). The final 3D models of the objects were displayed on a 2D monitor for the PC condition and in 3D via a head‐mounted display (HMD) in the VR condition. For the RL condition, all object pairs were printed using a 3D printer. The 40 originals from the identical pairs were printed twice to ensure that participants would not be able to determine whether the pairs of objects were identical or unidentical solely by taking individual characteristics of the texture resulting from the printing process into account. Hence, a total of 160 objects were printed with a maximum size of 10 cm^3^. The texture of the 3D objects in both the PC and the VR condition was aligned to the texture of the physical 3D prints. The viewing angle on the objects was calculated for the RL condition based on the largest object (10cm^3^). For this purpose, it was placed in the experimental setup and the distance to a seated viewer was measured, from which the viewing angle was calculated and resulted in a maximum vertical viewing angle of 7.01°. The viewing angle of both other conditions was matched by scaling the same object within the VR environment and on the screen respectively, and maintaining a distance of 65 cm across conditions. The remaining 80 objects were only included in the retrieval session as 2D renderings, i.e., planar pictures.

### 2.4 Setup and procedure

The experiment consisted of two phases, the encoding and the retrieval session, both including an EEG acquisition and carried out on the same day. The whole procedure took approximately 3.5 hours. The encoding session was based on a delayed matching‐to‐sample paradigm (for details see below), while the retrieval session included a remember/know recognition memory task. Since the publication at hand exclusively focuses on research questions and analyses based on the encoding session alone, the retrieval session will only be outlined briefly. The data obtained from the retrieval session are considered under a separate research objective and are thus addressed in a companion publication.

*Setup of the encoding session*. The encoding session was either carried out in real‐life (RL), via a PC monitor (PC) or via a VR‐HMD (VR). Participants were randomly assigned to one of these conditions, yet participants wearing glasses were randomly assigned to the RL or the PC condition due to technical constraints. The choice of a between design was particularly essential for the retrieval phase, which is discussed in detail in a companion paper (cf. methods, 2.0). In addition, we considered conducting three encoding sessions of approx. 60 minutes each plus the subsequent recall too strenuous for the participants.

**FIGURE 2:**
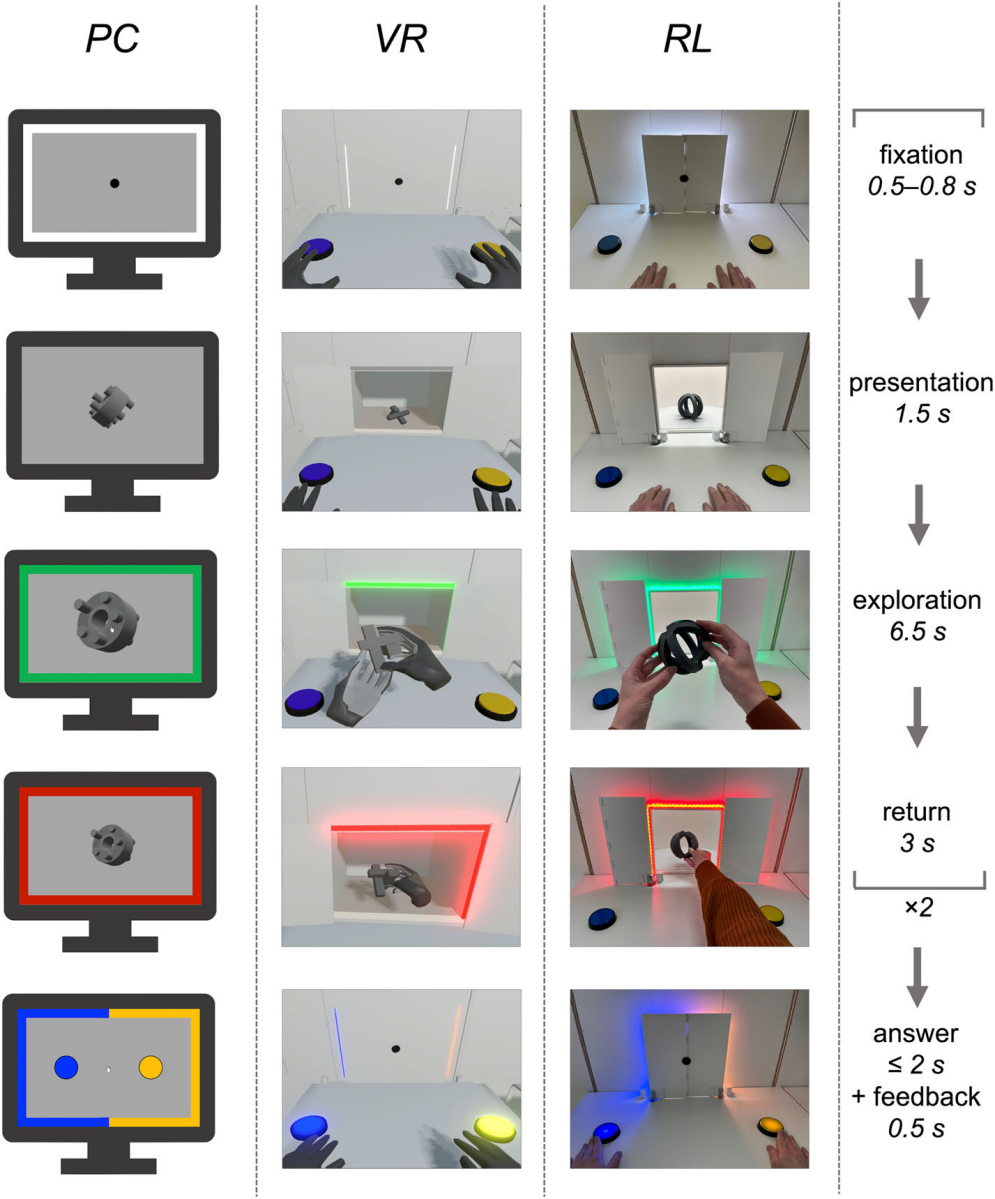
*Experimental procedure of the encoding session per experimental condition.* Participants performed a delayed matching‐to‐sample task. Each trial started with a fixation, followed by the presentation of an original object. Participants were subsequently instructed to explore the object and to return it as soon as the light flashed red. An ITI followed as a delay period during which the participants had to retain the original object. After the ITI, either a copy or variant of the original object was presented. After the stimulus offset, participants indicated via button press whether the object pair was identical or unidentical. Please note that this figure resembles the illustration of the experimental procedure of the companion papers to achieve the highest congruence and transparency between publications.

All three experimental conditions were constructed according to the same concept to maintain high comparability. Participants in all conditions wore earplugs to reduce external noise. Participants assigned to the RL condition were seated at a table facing the back of a shelf at a distance of 65 cm (see Figure 2). Two doors covered a window in the shelf's back and were connected to a stepper motor controlled by a microcontroller (Raspberry Pi4; Raspberry Pi Ltd, Cambridge, GB). During the experiment, the doors opened and closed automatically to display the objects placed behind them. The doors produced a sound of around 80 dB when opening and closing. Additionally, a light strip with 90 LEDs was placed around the window to provide a colour‐coded light signal, and a black fixation point was attached to the doors. The program controlling the doors and the LED strip was developed in Thonny (https://thonny.org; version 3.3.14) using Python (version 3.9.2). Two mechanical buttons were integrated into the table to register the participants' responses to their task during encoding (see procedure).

Both the PC and VR conditions were created in Unity (version 2020.3.3f1; Unity Technologies, San Francisco, USA) and aligned to the RL condition. In the VR condition, the laboratory environment and experimental setup of the RL condition were recreated (see Figure 2) and presented using an HTC Vive Pro 2 (HTC, Taoyuan, Taiwan) VR‐HMD with a resolution of 2448 × 2448 pixels per eye, 60° field of view and 90 Hz frame rate. The hand‐tracking device *Leap Motion Controller 1* (Ultraleap Limited, Bristol, England), was attached to the VR‐HMD allowing for interactions with the objects. The mechanical buttons, the LED strip, and the participants' seating position corresponded to those in the RL condition. The sound of the doors was recorded from the RL condition and integrated into the VR condition. It was played at 80 dB via the integrated headphones of the VR‐HMD.

In the PC condition, the objects were displayed two‐dimensionally on a conventional monitor (24″, 1920 × 1200 pixels resolution, 60 Hz frame rate) against a light grey background. A conventional computer mouse was used to interact with the objects. Participants were seated with 65 cm distance to the screen to match the viewing angle of both other conditions. Whenever responses were required, two 2D buttons as equivalents to the mechanical buttons were displayed on the screen to register the participants responses, and a coloured frame was displayed at the edges of the screen as an equivalent to the LED strip (see Figure 2). Doors in the same colour as the background were used as an equivalent to the real‐life doors to match the visual impression of the opening and closing process. The sound of the doors was played via speakers at 80 dB.

*Procedure of the encoding session*. After completing the anamnesis, the EEG was set up and the participants were either equipped with the VR‐HMD, seated in front of the 2D monitor in the PC condition, or seated at the table in the RL condition. They were instructed to perform a delayed matching‐to‐sample task, i.e., a pair of objects was subsequently presented with a short time delay during which the original object had to be retained. In more detail, participants were asked to initially only look closely at each object presented. They were asked to sit still and avoid blinking (Figure 2, fixation and presentation). After 1.5 s, they were asked to explore the object using their hands (RL, VR) or a computer mouse (PC; Figure 2., exploration & return). After the offset of the second object, participants indicated whether the pair of objects was identical or not (Figure 2, answer and feedback). The experiment started with a training session consisting of two trials, one with an identical object pair and one with an unidentical object pair. The aim of the training was to familiarize the participants with the procedure and the handling of the respective condition (e.g., interacting with the object via the hand‐tracking device). The objects presented during training were not used in the experimental trials. The training session could be repeated as often as required. After ensuring that the participants understood their task and were able to perform it without technical hurdles, the experimental trials were started. For each of the 80 trials, the colour‐coded signal provided information about the current phase of the trial. Each trial started with a white signal (5 s). As soon as the white signal went off, a fixation phase followed (randomly 0.5–0.8 s). Participants in the RL and the VR conditions were to fixate the dot attached to the closed doors, while a fixation dot appeared in the centre of the screen in the PC condition (see Figure 2: fixation). Afterwards, the doors opened (0.15 s to fully opened) and the participants were to look at the object placed behind the doors in a random rotation (1.5 s; see Figure 2: presentation). The start of the exploration phase was indicated by the onset of a green signal (6.5 s). In the RL condition, participants reached for the real‐life object and explored it with their hands, whereas interaction with the virtual 3D object in VR was realized by real‐time hand tracking (see Figure 2: exploration). In the PC condition, participants used the standard mouse to explore the 2D version of the object by means of zooming it in and rotating it on all three axes. After the exploration time, the colour‐coded signal started to flash in red (3 s). The objects had to be returned in the RL and the VR condition, and to be clicked with the computer mouse in the PC condition to zoom out the object (see Figure 2: return). After the doors were closed (0.65 s to fully closed), the colour‐coded signal turned white (5 s), indicating the inter‐trial‐interval (ITI). The object was exchanged by either the copy or the variant of the original object and presented following the same procedure. After the doors closed and covered the second object, the colour‐coded signal turned blue and yellow, the buttons in the RL and VR conditions lit up and the buttons appeared on the screen in the PC condition. Participants had to indicate whether the two explored objects were identical or not within 2 s (see Figure 2: answer). It was counterbalanced which button participants had to press to indicate that the objects were identical or unidentical. If participants did not answer fast enough or gave an incorrect answer, the colour‐coded signal turned off (0.5 s). In case the answer was correct, the colour‐coded signal flashed blue and yellow for 0.5 s instead. After 40 trials, all participants had a two‐minute break with the possibility to extend it if needed. Exemplary video material of the three encoding modalities can be found at OSF (please see data availability statement).

*Retrieval session*. The retrieval session will be examined in a companion paper and is thus outlined only briefly for the sake of completeness. The retrieval followed the encoding session directly after a change of room. Participants of all encoding conditions performed a remember/know recognition memory task on a conventional monitor (24″, 1920 × 1200 pixels resolution, frame rate of 60 Hz). In 160 trials, 80 2D pictures of the objects from the encoding session as well as 80 new objects were displayed. Participants had to indicate whether they remembered the object from the encoding session or not.

### 2.5 EEG recording

One‐hundred and twenty‐eight active electrodes by BioSemi (Amsterdam, Netherlands) were attached in accordance with the international 10–20‐system. Additionally, a Common Mode Sense (CMS) and a Driven Right Leg (DRL) electrode were used as ground and reference electrodes. An electrooculogram (EOG) was obtained using four electrodes attached around the eyes. The data were recorded at a sampling rate of 512 Hz and online‐filtered at 0.016–100 Hz. During the encoding session, LabRecorder was used to record the EEG data stream and synchronize it with the triggers sent by Lab Streaming Layer (LSL by SCCN, https://github.com/sccn/labstreaminglayer). During the retrieval session, EEG was recorded using ActiView702 (BioSemi, Amsterdam, Netherlands).

### 2.6 EEG preprocessing

**FIGURE 3:**
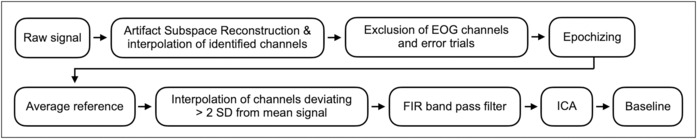
*Schematic flowchart of the preprocessing pipeline.* The details on each step are described in section *2.6* *EEG preprocessing*.

All analyses in this publication refer to the static presentation of the object (see Figure 2, *presentation*). During this time window, the participants were instructed not to move or blink. Interactions with the object took place in the subsequent sections (see Figure 2, *exploration, return, answer*). A schematic, condensed overview of the preprocessing steps can be found in Figure 3. The preprocessing steps were kept the same across all companion papers to ensure comparability of the data (see methods, 2.0).

The EEG data were preprocessed using Matlab (version R2023a, MathWorks Inc.) and EEGLAB (version 2023.0, Delorme & Makeig, 2004). As a first step, bad channels were identified using Artifact Subspace Reconstruction (ASR; default settings; Mullen et al., 2015). On average, 0.74 channels were interpolated (*SD* = 2.12; *M*_RL_ = 0.46, *M*_VR_ = 1.52, *M*_PC_ = 0.27). The EOG channels were excluded from all further preprocessing steps. The latency of the event triggers sent via LSL during data acquisition was counterchecked and corrected using a photodiode. Additionally, trials in which an error occurred during the encoding session were excluded (e.g., if the objects were not correctly displayed). This applied to only 10 datasets, for which an average of 1.8 trials were excluded (*M*_RL_ = 0.21, *M*_VR_ = 0.33, *M*_PC_ = 0). The data were epochized from −500 ms before the door started opening to 1500 ms afterwards. The epochized data were re‐referenced to average reference. Channels deviating by more than two standard deviations from the mean of all electrodes were identified and interpolated (*M* = 5.39, *SD* = 1.82; *M*_RL_ = 5.76, *M*_VR_ = 5.73, *M*_PC_ = 4.70). A FIR band pass filter from 0.25 Hz to 30 Hz and linear detrending were applied. An independent component analysis (ICA) was used to detect and remove artefacts classified as eye (0.8 probability), muscle, heart, channel noise and line noise (0.9 probability each) by the IClabel function (Pion‐Tonachini et al., 2019). The IClabel function excluded 1.63 components on average (*SD* = 1.26; *M*_RL_ = 1.94, *M*_VR_ = 1.42, *M*_PC_ = 1.52). Moreover, the data were manually checked for remaining eye‐related artefacts. A baseline of −500 to −300 ms before the door opening was applied.

To analyse the frequency domain, a Morlet wavelet analysis was conducted (see Bertrand & Pantev, 1994; Cohen, 2014). The wavelet width was set to archive an appropriate frequency resolution of 0.5 Hz, resulting in 199 wavelets ranging from 1 to 100 Hz (for a detailed description of the procedure please see Bertrand & Pantev, 1994). Both the evoked and induced oscillatory activities were taken into account. The evoked oscillatory activity was based on the data averaged across trials in the time domain prior to the frequency decomposition. Yet the induced oscillatory activity tends to cancel out if trials are averaged due to a jitter in latency after stimulus onset (Eckhorn et al., 1990). Hence, to allow for analysis of the non‐phase‐locked components, the evoked response, i.e., the ERP, was subtracted from each trial before the frequency decomposition was conducted. The time‐frequency (TF) amplitudes were thus averaged over the frequency transformations of a single trial, resulting in non‐phase‐locked oscillatory, i.e., induced activity. This procedure ensures that the analyses of induced activities are independent of the phase‐locked processes reflected in the evoked oscillatory activity (see Busch et al., 2006; Gruber et al., 2004; Kisker et al., 2024 for a similar procedure). After averaging the trials, a baseline of −300 to 0 ms before stimulus onset was applied per dataset.

**FIGURE 4:**
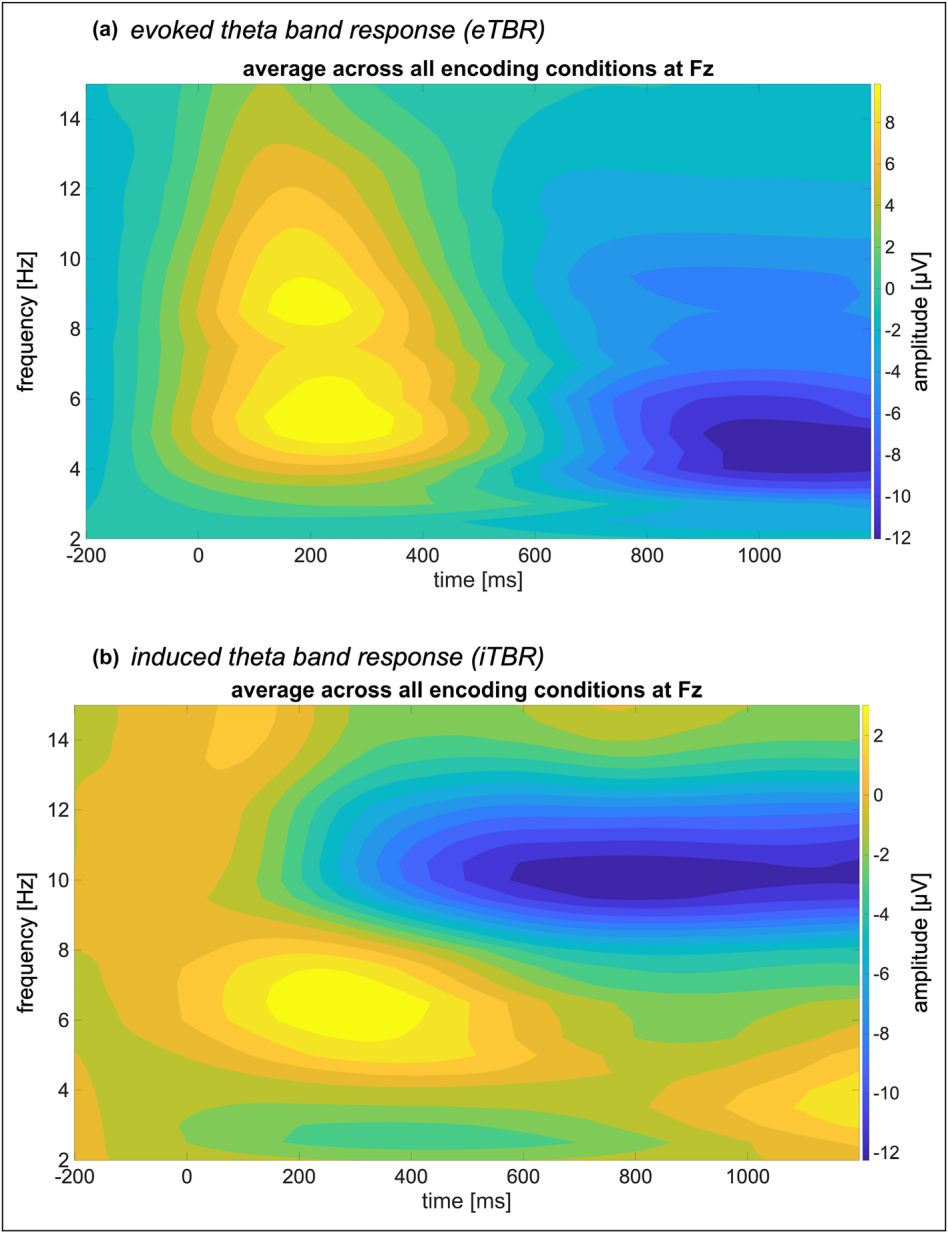
*Time‐frequency (TF) representation of the evoked and induced theta band responses measured at Fz.* The TF‐plot visualizes [A] the evoked and [B] the induced theta band response averaged across all conditions from 2 to 15 Hz as measured at the electrode Fz. For the eTBR, the range from 4 to 7.5 Hz was included in the analyses, while the range from 5 to 8 Hz was chosen for the iTBR. Albeit the TF‐plot indicates a pronounced amplitude increase in the evoked alpha band range (approx. 8–10 Hz), this range was not included in the analyses as it goes beyond the research objective and related hypotheses at hand.

The frequency ranges and electrodes of interest were derived as regions of interest from previous literature and adapted by means of a visual inspection of the time‐frequency (TF)‐plots, depicting the magnitude of the data per individual frequency band varying over time, and topography of the grand mean across all conditions of the induced and evoked oscillations respectively. For the eTBR, the frequency range from 4 to 7.5 Hz was chosen. The TF‐plot indicated a pronounced increase in the alpha band range (approx. 8–10 Hz) as well. Yet the latter was not included in analyses since it goes beyond the research question and related hypotheses at hand. Since the alpha band response is assumed to contribute to the generation of the P1‐N1 complex (Klimesch et al., 2004, 2007), the increased alpha amplitude after stimulus onset might reflect the ERP response after stimulus onset, which is subtracted from the total response when calculating the induced response. It would therefore be no longer visible in the induced response (see Figure 4). Parietal electrodes around Pz and posterior electrodes around Oz were chosen as separate clusters to replicate the analyses by Tang et al. (2022). The visual inspection of the mean topography revealed a strong amplitude distribution around Cz as well, while no distinct distribution was observed for the parietal cluster adopted by Tang et al. (2022). For that reason, electrodes surrounding Cz were chosen as an additional electrode cluster for analyses. The time window from stimulus onset to 300 ms after stimulus onset was adopted from Tang et al. (2022) and complemented by a later time window from 300 to 600 ms after stimulus onset. Both time windows were counterchecked against the TF representation of the data. For the iTBR, the range from 5 to 8 Hz was chosen and an electrode cluster including Cz and Fz was chosen similar to related studies focusing on midfrontal theta (Li et al., 2020; Slobounov et al., 2015; Xu & Sui, 2021). Per visual inspection, an additional posterior cluster surrounding Oz was included in the analyses (for a similar procedure see Kisker et al., 2024). Based on previous studies, three‐time windows with a duration of 250 ms ranging from stimulus onset to 750 ms after stimulus onset were included in the analyses (e.g., Klimesch et al., 1997). All time windows were counterchecked against the TF representation of the data to ensure that the analyses would include the respective activity of interest (see Figure 4). A baseline from 300 ms before stimulus onset until stimulus onset was applied prior to statistical analyses.

### 2.7 Statistical analyses

The responses given during the delayed matching‐to‐sample task were compared between groups by means of a one‐way ANOVA and followed by post‐hoc *t*‐tests for independent samples. The number of correct answers was relativized to the actual answers given in order to account for potential differences in the number of omissions (e.g., due to the novelty of the hand‐tracking device).

The induced and evoked theta band responses were analysed using mixed ANOVAs and post‐hoc *t*‐tests for independent samples for the respective group comparisons. The differentiation of the first presentation and the second presentation of each object was not of interest to the research objective at hand. Consequently, a preceding mixed ANOVA including the factors PRESENTATION, TIMING and GROUP was applied to check for any main effects or interactions of the within‐factor presentation. In case the ANOVA indicated a main effect of, or interaction with the factor presentation, only the responses to the first presentation were further analysed to prevent the influence of possible habituation or sensitization. Otherwise, the data were averaged across the first and second presentations for further analyses.

For the evoked theta band response (**e**TBR), 2 × 2 × 3 mixed ANOVAs covering the within‐factors PRESENTATION (first, second) and TIMING (0–300 ms, 300–600 ms), and the between‐factor GROUP (PC, VR, RL) were calculated for a central, a parietal and a posterior electrode cluster. The eTBR to second presentations was excluded from analyses due to a significant main effect of the factor PRESENTATION and related interactions (see supplementary material S1 ‐ S3). Consequently, a 2 × 3 mixed ANOVA covering the within‐factor TIMING (0–300 ms, 300–600 ms) and the between‐factor GROUP (PC, VR, RL) was calculated for the eTBR to first presentations. If the Mauchly test indicated violations of sphericity, the *F*‐values were Greenhouse–Geisser corrected.

Congruently, 2 × 3 × 3 mixed ANOVAs covering the within‐factors PRESENTATION (first, second) and TIMING (0–250 ms, 250–500 ms, 500–750 ms), and the between‐factor GROUP (PC, VR, RL) were calculated for a midfrontal and a posterior electrode cluster for the induced theta band response (**i**TBR). Due to a significant main effect of the factor PRESENTATION and related interactions (see supplementary material S4 ‐ S5), the iTBR to second presentations were excluded from analyses. Accordingly, 3 × 3 mixed ANOVAs covering the within‐factor TIMING (0–250 ms, 250–500 ms, 500–750 ms) and the between‐factor GROUP (PC, VR, RL) were only calculated for the iTBR to first presentations per cluster.

When the mixed ANOVAs indicated respective main effects and interactions, post‐hoc *t*‐tests were performed by means of planned comparisons. The comparison of the groups was of particular interest but not the comparison of the time windows. If the Levene test for equality of variances indicated significantly different variances, the corrected *t*‐tests were reported. The inferential statistics were complemented by the calculation of the Bayes factor (BF) for each post hoc *t*‐test to allow for more robust conclusions about the differences between the groups, and importantly, about potential similarities between groups. The BF for independent samples was calculated using RStudio (Posit PBC, Boston, USA). The *ttestBF* function from the *BayesFactor* Package (version 0.9.12–4.7; Morey & Rouder, 2011; Rouder et al., 2009) was applied using its default priors. A BF > 1 favours the H1, whereas a BF < 1 favours the H0.

## 3 RESULTS

### 3.1 Discrimination performance

The number of given answers or omitted answers respectively differed between groups (*F*[2,96] = 11.76, *p* < .001; omissions: *M*_PC_ = 0.91, *SD*_PC_ = 1.77; *M*_VR_ = 3.00, *SD*_VR_ = 3.92; *M*_RL_ = 0.12, *SD*_RL_ = 0.33). Specifically, the VR group omitted more answers than the PC group (*t*[44.58] = −2.79, *p* = .008; *d* = 0.69) and the RL group (*t*[32.46] = −4.20, *p* < .001; *d* = −1.04), while the PC group omitted more answers than the RL group (*t*[34.23] = 2.51, *p* = .017; *d* = −0.62). These omissions can at least partwise be traced back to the different interaction styles, since participants had to get used to interacting via the motion tracker in the VR condition, whereas the RL interactions were fully natural (see 2.4). Yet participants performed equally well in this task: 65% of the answers given were correct, without significant differences between groups (*F*[2,96] = 1.38, *p* = .256; *M*_PC_ = 0.64, *SD*_PC_ = 0.06; *M*_VR_ = 0.66, *SD*_VR_ = 0.06; *M*_RL_ = 0.65, *SD*_RL_ = 0.07).

### 3.2 Evoked theta band response (eTBR)

#### 3.2.1 Central cluster

For the central cluster, the 2 × 3 mixed ANOVA indicated a significant main effect of the factor TIMING (*F*[1, 96] = 116.55; *p* < .001; *η* ^*2*^ = .55), no significant main effect of the factor GROUP (*F*[2,96] = 0.29; *p* = .075; *η* ^*2*^ = .01) and a significant interaction of both factors (*F*[2, 96] = 9.29; *p* < .001; *η* ^*2*^ = .16). Yet the eTBR did not differ between groups for either time window (all *ts*[64] < |1.27|; all *ps* > .05; see Table 1). In line with this, the BFs for these comparisons moderately to anecdotally favoured the H0 (see Table 1).

**TABLE 1:** Test statistics for the post‐hoc tests of the eTBR.

		*Two‐tailed t‐test for independent samples*				*Bayesian stats*	
		*t*	*Df*	*p*	*d*	*BF*	*Favours*
Central eTBR 0–300 ms	PC vs. VR	1.05	64	.296	0.26	0.40^e^	H0
	PC vs. RL	−0.19	64	.850	−0.05	0.26^d^	H0
	VR vs. RL	−1.27	64	.210	−0.31	0.50^e^	H0
Central eTBR 300–600 ms	PC vs. VR	−0.05	64	.961	−0.01	0.25^d^	H0
	PC vs. RL	1.60	64	.115	0.39	0.74^e^	H0
	VR vs. RL	1.74	64	.086	0.43	0.90^e^	H0
Parietal eTBR 0–300 ms	PC vs. VR	−1.87	64	.067	−0.46	1.09^e^	H1
	PC vs. RL	−0.97	64	.334	−0.24	0.38^e^	H0
	VR vs. RL	1.17	64	.245	0.29	0.45^e^	H0
Parietal eTBR 300–600 ms	PC vs. VR	3.63	64	.001	0.89	49.43^b^	H1
	PC vs. RL	−0.12	64	.908	−0.03	0.25^d^	H0
	VR vs. RL	−4.37	64	<.001	−1.07	429.26^a^	H1
Posterior eTBR 0–300 ms	PC vs. VR	−0.82	64	.416	−0.20	0.34^e^	H0
	PC vs. RL	−2.97	48.22	.005	−0.73	9.38^d^	H1
	VR vs. RL	−1.96	64	.054	−0.48	1.27^e^	H1
Posterior eTBR 300–600 ms	PC vs. VR	−2.10	64	.039	−0.52	1.60^e^	H1
	PC vs. RL	−4.54	64	<.001	−1.12	730.99^e^	H1
	VR vs. RL	−2.15	64	.035	−0.53	1.74^e^	H1

*Note*: The table contains the inferential statistical parameters of the two‐sided *t*‐tests for independent samples, Cohen's effect size *d* and the Bayes factor (BF) for each comparison regarding the eTBR. A BF > 1 favours the H1, a BF < 1 favours the H0. Commonly used thresholds of the magnitude of evidence by the BF are indicated as follows: a) extreme, b) very strong, c) strong, d) moderate and e) anecdotal (e.g., Jeffreys, 1998).

#### 3.2.2 Parietal cluster

**FIGURE 5:**
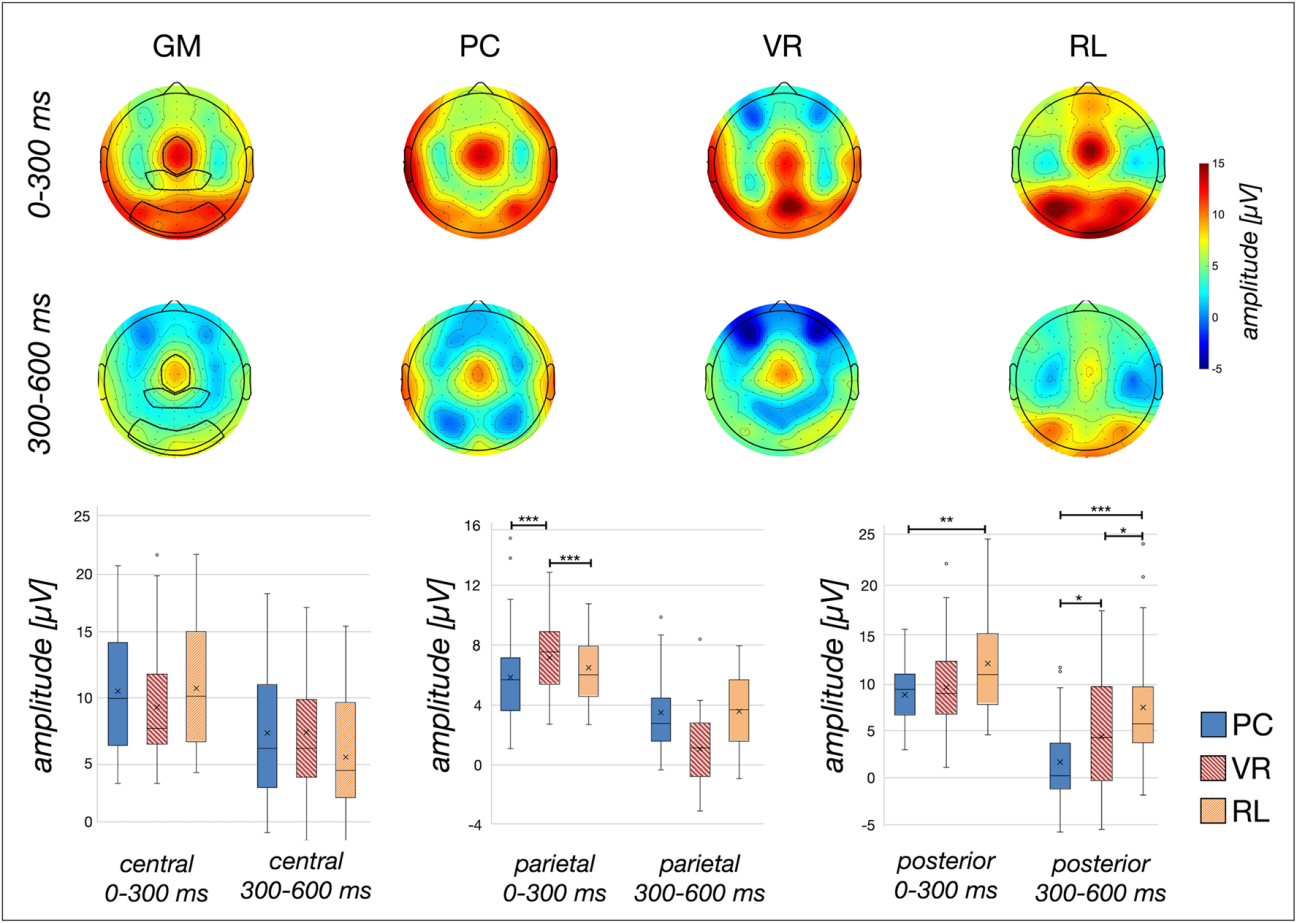
*Visualization of the evoked oscillatory response in the theta band range from 4 to 7.5 Hz.* The amplitude distribution is depicted separately for each analysed time window for the average across conditions (GM), as well as per condition. The electrodes used for statistical comparisons are marked in the respective mean topographies: the central cluster includes eleven electrodes including and neighbouring Cz, the parietal cluster includes CP3, CP1, CPZ, CP2, CP4, P3, P1, PZ, P2, P4 (adopted from Tang et al., 2022) and five neighbouring electrodes, and the posterior cluster includes O1, O2, Oz (adopted from Tang et al., 2022) and twelve neighbouring electrodes. The box plots indicate the mean amplitude for the indicated electrode cluster and time window. Significant differences are marked respectively: *p < .05, **p < .01, ***p < .001.

For the parietal cluster, the 2 × 3 mixed ANOVA indicated a significant main effect of the factor TIMING (*F*[1, 96] = 231.59; *p* < .001; *η* ^*2*^ = .71), no significant main effect of the factor GROUP (*F*[2, 96] = 1.24; *p* = .29; *η* ^*2*^ = .03) and a significant interaction of both factors (*F*[2, 96] = 27.35; *p* < .001; *η* ^*2*^ = .36). The post‐hoc *t*‐tests revealed no difference in the eTBR between the PC group and the RL group during either time window (all *ts*[64] < |0.97|; all *ps* > .30). Yet post hoc *t*‐tests indicated a significantly lower eTBR to the VR compared to the PC group (*t*[64] = 3.63; *p* < .001; *d* = 0.89) and the RL group (*t*[64] = −4.37; *p* < .001; *d* = −1.08; see Figure 5) during the later time window from 300 ms to 600 ms. During the early time window, no significant differences between VR and both the PC group and the RL group were revealed (all *ts*[64] < |1.87|; all *ps* > .06; see Table 1). The BF favoured the H1 very strongly to extremely for comparisons that were inferentially statistically significant and moderately to anecdotally the H0 for differences that were not inferentially statistically significant (see Table 1).

#### 3.2.3 Posterior cluster

For the posterior cluster, the 2 × 3 mixed ANOVA indicated a significant main effect of the factor TIMING (*F*[1, 96] = 216.19; *p* < .001; *η* ^*2*^ = .69), a significant main effect of the factor GROUP (*F*[2,96] = 8.09; *p* < .001; *η* ^*2*^ = .14) and a significant interaction of both factors (*F*[2, 96] = 3.98; *p* < .022; *η* ^*2*^ = .08). During the time window from 0 ms to 300 ms, the PC group and the RL group yielded significantly different eTBRs, with a higher eTBR in the RL group (*t*[48.22] = −2.97; *p* = .005; *d* = −.73 see Figure 5). Yet no significant difference was revealed between the VR group and both other groups (all *ts*[64] < |2.15|; all *ps* > .05). During the time window from 300 ms to 600 ms, all groups yielded significantly different eTBRs with the highest eTBR in the RL group and the lowest eTBR in the PC group (all *ts*[64] > |2.10|; all *ps* < .05; all *ds* > − .51; see Table 1 and Figure 5). Consistent with these tests, the BF favoured the H1 for comparisons that were inferentially statistically significant and the H0 for differences that were not inferentially statistically significant (see Table 1). The BF indicated anecdotical evidence for these respective comparisons, with the exception of the comparison of the PC and RL condition, for which the BF indicated very strong to extreme evidence for the H1.

### 3.3 Induced theta band response (iTBR)

#### 3.3.1 Midfrontal cluster

**FIGURE 6:**
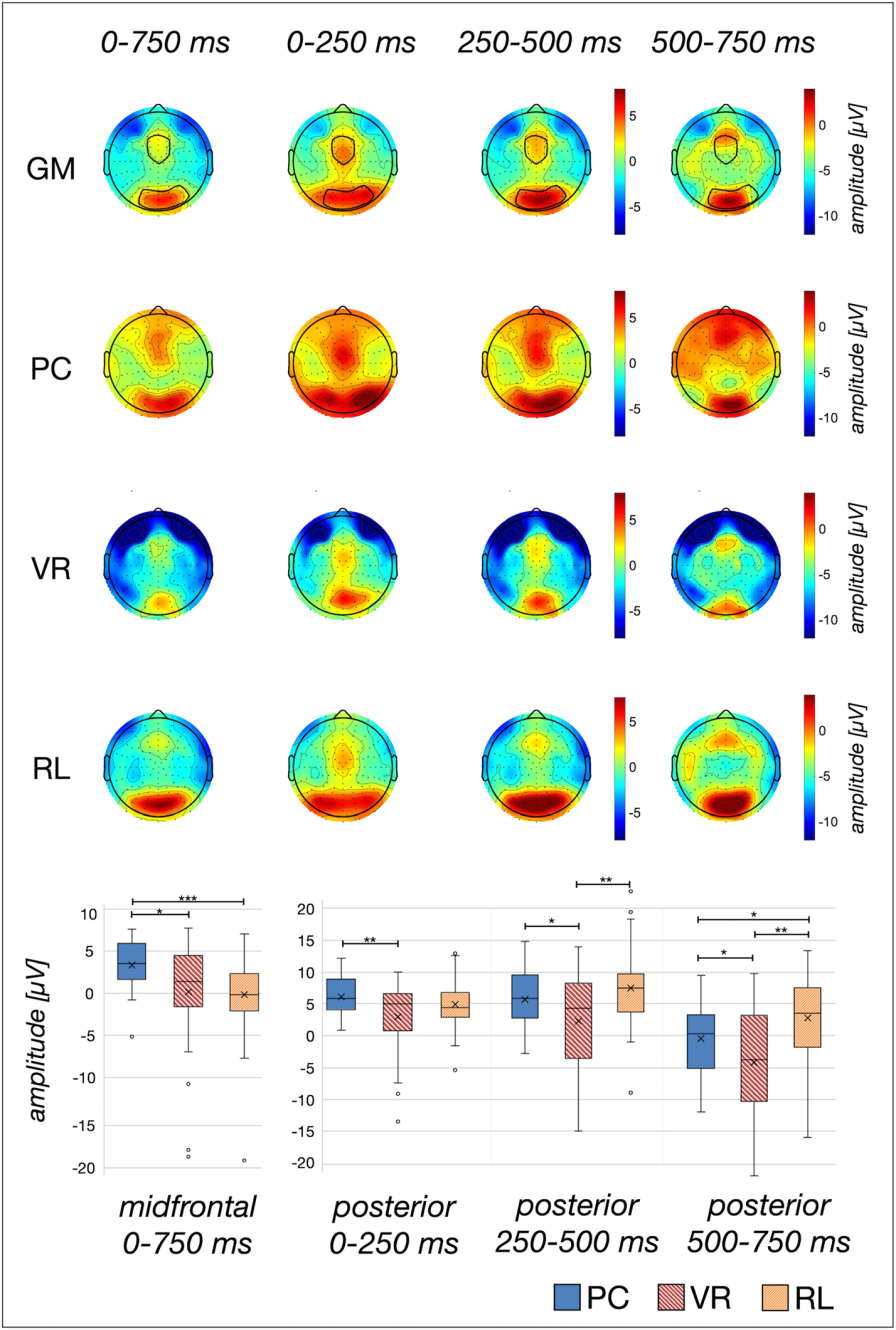
*Visualization of the induced oscillatory response in the theta band range from 5 to 8 Hz.* The amplitude distribution is depicted separately for each analysed time window for the average across conditions (GM), as well as per condition. The electrodes used for statistical comparisons are marked in the respective mean topographies: the midfrontal cluster includes Cz, Fz, FC1, FC2, F1, F2 and six neighbouring electrodes. The posterior cluster includes O1, Oz, O2, Pz, PO3, PO4, PO7, PO8 and four neighbouring electrodes. The box plots indicate the mean amplitude for the indicated electrode cluster and time window. Significant differences are marked respectively: *p < .05, **p < .01, ***p < .001.

The 3 × 3 mixed ANOVA revealed a significant main effect of the factor TIMING (*F*[1.31, 126.18] = 189.79; *p* < .001; *η* ^*2*^ = .66), a significant main effect of the factor GROUP (*F*[2, 96] = 5.25; *p* = .007; *η* ^*2*^ = .10), but no significant interaction of both factors (*F*[2.63, 126.18] = 1.49; *p* = .223; *η* ^*2*^ = .03). Consequently, the data were averaged across the factor TIMING to further analyse the main factor GROUP by means of planned contrasts. Post‐hoc *t*‐tests revealed a significantly higher iTBR in the PC group compared to the VR group (*t*[43.95] = 2.61; *p* < .012; *d =* 0.64), as well as compared to the RL group (*t*[64] = 3.69; *p* < .001; *d =* 0.91). In contrast, no significant difference was found for the iTBR to the VR group and the RL group (*t*[64] = 0.23; *p* = .818; see Table 2 and Figure 6). Congruent with these *t*‐tests, the BF favoured the H1 moderately to strongly for comparisons that were inferentially statistically significant, and the H0 moderately for differences that were not inferentially statistically significant (see Table 2).

**TABLE 2:** Test statistics for the post‐hoc tests of the iTBR.

		*Two‐tailed t‐test for independent samples*				*Bayesian stats*	
		*t*	*Df*	*p*	*d*	*BF*	*Favours*
Midfrontal iTBR 0–750	PC vs. VR	2.61	43.95	.012	0.64	4.25^d^	H1
	PC vs. RL	3.69	64	<.001	0.91	58.48^b^	H1
	VR vs. RL	0.23	64	.818	0.06	0.26^d^	H0
Posterior iTBR 0–250 ms	PC vs. VR	2.86	50.24	.006	0.70	7.26^d^	H1
	PC vs. RL	1.44	64	.155	0.35	0.60^e^	H0
	VR vs. RL	−1.63	64	.108	−0.40	0.77^e^	H0
Posterior iTBR 250–500 ms	PC vs. VR	2.08	49.32	.043	0.51	1.53^e^	H1
	PC vs. RL	−1.37	64	.175	−0.34	0.56^e^	H0
	VR vs. RL	−2.92	57.96	.005	−0.72	8.41^d^	H1
Posterior iTBR 500–750 ms	PC vs. VR	2.06	54.39	.044	0.51	1.49^e^	H1
	PC vs. RL	−2.19	64	.032	−0.54	1.87^e^	H1
	VR vs. RL	−3.71	64	<.001	−0.91	61.88^b^	H1

*Note*: The table contains the inferential statistical parameters of the two‐sided *t*‐tests for independent samples, Cohen's effect size *d* and the Bayes factor (BF) for each comparison regarding the iTBR. A BF > 1 favors the H0, a BF < 1 favors the H1. Commonly used thresholds of the magnitude of evidence by the BF are indicated as follows: a) extreme, b) very strong, c) strong, d) moderate, e) anecdotal (e.g., Jeffreys, 1998).

#### 3.3.2 Posterior cluster

Regarding the posterior electrode cluster, the 3 × 3 mixed ANOVA indicated a significant main effect of the factor TIMING (*F*[1.37, 131.68] = 126.14; *p* < .001; *η* ^*2*^ = .57), a significant main effect of the factor GROUP (*F*[2, 96] = 6.22; *p* = .003; *η* ^*2*^ = .12), as well as a significant interaction of both factors (*F*[2.74, 131.68] = 8.31; *p* < .001; *η* ^*2*^ = .15). The planned post‐hoc *t*‐tests indicated significantly higher iTBRs to the PC group compared to the VR group across all time windows (all Levene‐corrected *t*s > 2.06; all *p*s < .044, all *d*s > 0.5, see Table 2). Yet the iTBR of the PC group differed from the RL group during the latest time window from 500 ms to 750 ms (*t*[64] = −2.19; *p = *.032, *d = −*0.54), but no significant difference was revealed during both earlier time windows (both *t*s <|1.50|; both *p*s > .10, see Table 2). Last but not least, the iTBR of the VR group did not differ significantly from the RL group from 0 ms to 250 ms (*t*[64] = −1,63; *p = *.108) but during both consecutive, later time windows (both Levene‐corrected *t*s >|2.90|; both *p*s <.05), revealing a higher iTBR in the RL group compared to the VR group (see Table 2 and Figure 6). In line with the *t*‐tests, the BF favoured the H1 for comparisons that were inferentially statistically significant and the H0 for differences that were not inferentially statistically significant (see Table 2). The BF indicated anecdotical to moderate evidence for these comparisons, with the exception of posterior iTBR in the latest time window compared between VR and RL, for which the BF indicated very strong evidence for the H1.

## 4 DISCUSSION

### 4.1 Summary

The study at hand examines the differences in visual information processing of conventional 2D objects, virtual 3D objects and real‐world 3D objects. We aimed to differentiate between responses predominantly driven by sensory stimulus features mirrored in the evoked theta band response (**e**TBR), and more complex cognitive processes reflected in the induced theta band response (**i**TBR). To this end, participants were presented with abstract objects in a delayed matching‐to‐sample task using either 2D objects presented on a conventional desktop (PC), 3D objects presented in virtual reality (VR), 3D objects presented in a real‐world setting (RL) allowing for a triangular comparison. Albeit all groups achieved the same proportion of correct answers in this task, pronounced differences were found in the electrophysiological markers of visual information processing. In particular, the posterior eTBR differentiated not only binarily in terms of 2D and 3D stimuli but between all conditions. Whereas fewer differences were revealed between the perception of objects in VR and RL compared to PC, the specific pattern of results indicated that visual information beyond three‐dimensionality contributes to the differentiation of these conditions. Even more interestingly, the midfrontal iTBR indicated higher cognitive load during visual processing of 2D objects compared to both kinds of 3D objects, while VR and RL did not significantly differ in the midfrontal iTBR. In contrast, the differentiation by means of the posterior iTBR increased over time, distinguishing all conditions in the latest analysed time window with real‐world 3D objects exhibiting the highest response. Yet the functional role of the posterior iTBR needs further examination.

### 4.2 Evoked theta band responses mirror visuospatial processing beyond three‐dimensionality

Albeit the differentiation of the neural responses to the original objects and either their copy or their variant goes beyond the research question at hand, the participants' performance in the delayed matching‐to‐sample task has relevant implications for the visual information processes at work during object presentation: Participants had to determine whether a pair of subsequently presented objects was identical (i.e., original and copy) or unidentical (i.e., original and variant). All groups achieved an accuracy of approximately 65% in this task. With only 15% above the chance level, the task was not particularly easy to solve, which generally indicates that the participants were cognitively challenged and that a considerable amount of attention was required to solve the task. Although the groups omitted different rates of responses on average, this seemed to be related to technical aspects such as the hand tracking device rather than to a lack of decision‐making. Hence, more importantly, all groups were equally able to distinguish between identical and unidentical object pairs, implicating that relevant details have been perceived and maintained well enough to reach equal performance under all conditions. The differences found in the electrophysiological response can thus not be explained by variance in the task performance but can be attributed to differences in the underlying cognitive processes.

As a first step, we aimed to replicate and extend the findings by Tang et al. (2022), proposing that the theta band response (TBR) measured at parietal and posterior sensors differentiates 2D and 3D stimuli by means of a higher TBR to 3D materials. However, our results did not indicate that the eTBR would differentiate 2D and 3D stimuli by means of two binary categories. In the first 300 ms after stimulus onset, the eTBR measured at posterior sensors was significantly higher for real‐world 3D objects compared to 2D objects. This finding indicates that the initial response to a visually perceived stimulus depends to a large degree on the mode of presentation. Yet no difference was revealed comparing the VR objects to both other conditions, seemingly adopting intermediate processes between the visual perception of planar and real objects. In the subsequent time window from 300 to 600 ms, however, VR objects exhibited a significantly lower eTBR measured at parietal sensors than both other conditions, while the posterior cluster differentiated between all conditions. Across both time windows, the posterior eTBR was lowest for 2D stimuli and highest for real‐world 3D stimuli. Thus, even though occurring during a later time window than reported by Tang et al. (2022), the eTBR differentiated by means of the stimuli's dimensionality. Yet going beyond previous findings, the response differentiated between VR and real‐world stimuli as well. Consequently, real‐world 3D objects either create an even stronger impression of three‐dimensionality driving a higher eTBR compared to 3D objects in VR, or the response is driven by additional sensory features, negotiating a binary differentiation into 2D and 3D materials by means of the eTBR. However, the differences found were of quantitative, rather than qualitative nature. Hence, the eTBR might still indicate that the same visuospatial process is at work under all conditions but shows adaption by means of magnitude.

A binary differentiation into 2D and 3D processing is further undermined by the absence of a congruent response pattern when taking all electrode clusters and time windows into account: The extent to which the eTBR differentiates between the three conditions depends on the region and timing of interest. Yet the eTBR to the passive viewing of 2D and 3D materials has been associated not only with the degree or extent of three‐dimensionality but to attentional and mnemonic processing as well: As Malik et al. (2015) argue, 3D materials provide an overall higher amount and detail of visuospatial information that need to be processed, and are hence increasing attentional involvement and mnemonic processes compared to planar materials. Albeit we matched the experimental conditions in visual features like the stimuli's size, the visual angle and the stimuli's visual texture, the real‐world 3D objects are more sophisticated in some features, e. g., in terms of naturalness of shades or more fine‐grained textures. Yet if attentional and mnemonic processing differs between the three modes of stimulus presentation, these differences should be mirrored in complex cognitive processes reflected in the iTBR.

### 4.3 Lower cognitive load for 3D stimulus processing as mirrored in the induced theta band response

The midfrontal iTBR has been discussed as a marker for cognitive load (Dan & Reiner, 2017;Li et al., 2020; Slobounov et al., 2015). Hence, most importantly, cognitive load as mirrored by the midfrontal iTBR during visuospatial processing of either virtual 3D objects or real‐world 3D objects was not significantly different, indicating that both would lead to the same theoretical conclusions about the underlying processes to be drawn, while PC conditions might significantly over‐ or underestimate the cognitive load of processing their real‐world equivalents.

In more detail, our results support the notion that cognitive load is higher during visuospatial processing of 2D stimuli compared to both kinds of 3D stimuli (Dan & Reiner, 2017). Since the visual system is adapted to the continuous processing of a three‐dimensional environment, it seems plausible to assume that it does so without consuming a great amount of the cognitive resources available for continuous default tasks (see e.g., Dan & Reiner, 2017; Johnsdorf et al., 2023; Marini et al., 2019; Snow & Culham, 2021). With respect to findings proposing the opposite effect, namely cognitive load being higher during three‐dimensional VR experiences compared to PC conditions (Li et al., 2020; Slobounov et al., 2015), cognitive load might be modulated not only by the mere spatial features of the experimental stimuli but by the participants' tasks as well. Since our participants performed a delayed matching‐to‐sample task, the task's affordances might resemble the task implemented by Dan and Reiner (2017) most. Participants of this study learned to fold an origami figure either by watching a 2D or a 3D video respectively. Hence, participants of both studies had to process and maintain detailed visuospatial information about the configuration of the objects. In contrast, route learning tasks (Slobounov et al., 2015) and selective attention tasks (Li et al., 2020) yielded higher cognitive load under VR conditions, albeit this increased cognitive load yielded beneficial effects on learning compared to a 2D condition. Consequently, whether cognitive load is higher or lower during 3D conditions compared to 2D conditions seems to depend on the affordances of the specific task as well. Yet during visuospatial processing, cognitive load seems to be lower during VR and real‐world 3D conditions compared to the PC conditions.

An alternative explanation is offered by studies linking increases in the midfrontal iTBR to mnemonic encoding (see Hsieh & Ranganath, 2014 for a review). With respect to this interpretation, our results would indicate a more profound mnemonic processing of the 2D condition compared to both 3D conditions. Since participants needed to maintain detailed knowledge about the configuration of the presented original object and compare it to a subsequently presented copy or variant, the iTBR might mirror working memory processes in this setup (Gregory et al., 2022; Hsieh & Ranganath, 2014; Jaiswal et al., 2010; Jensen & Tesche, 2002; Klimesch, 1999). However, the performance of the delayed matching‐to‐sample task did not indicate differences in discriminatory performance that might result from distinct working memory processes. Consequently, either the iTBR does not specifically relate to working memory processes in this experimental setup, or, as previously argued in a study comparing the neural correlates of episodic memory retrieval after PC‐based compared to VR‐based encoding, the PC condition afforded more effortful mnemonic processing to achieve the same performance in the behavioural task (Kisker et al., 2021) which vice versa would score a point for higher cognitive load in the 2D condition.

Moreover, the interpretation of the iTBR mirroring mnemonic processing in this task conflicts with the reported results regarding the eTBR: The posterior eTBR indicates more sophisticated processing of both 3D conditions compared to the 2D condition, and distinguished between virtual 3D and real‐world 3D objects as well. In contrast, the midfrontal iTBR was highest for 2D objects and did not reveal a difference between both kinds of 3D objects. Hence, the posterior eTBR and midfrontal iTBR seem to reflect functionally different processes mirrored by the different patterns of results. However, the evaluation of the mnemonic processes at work remains of a speculative nature, while the interpretation of the iTBR mirroring cognitive load during visual information processing in both 2D and 3D settings is more profoundly supported by previous findings (Dan & Reiner, 2017; Li et al., 2020; Slobounov et al., 2015).

While the iTBR revealed a stringent pattern at midfrontal sensors, the exploratory analyses of the posterior amplitude distribution revealed differences between conditions depending on the timing after stimulus onset. Directly after stimulus onset, virtual 3D objects yielded a significantly lower iTBR compared to 2D objects, subsequently differing from real‐world 3D objects as well, and in the latest time window, the posterior iTBR differed between all conditions. If real‐world objects are initially kept aside, a differentiation between 2D and 3D stimuli similar to the results reported by Tang et al. (2022) is generally evident in the earliest time window, albeit the response is higher to 2D objects. A comparable posterior peak in the iTBR was observed in a previous study using virtual 3D objects in a repetition priming task and was discussed to be linked to the three‐dimensional presentation mode in VR (Kisker et al., 2024). Going one step further, our results reveal that the posterior iTBR is generally not unique to VR conditions. Moreover, the posterior iTBR is higher for 2D than for virtual 3D objects and the response to virtual 3D objects is not intermediate between the PC and real‐world conditions, but lowest. Consequently, the distinct posterior iTBRs do not seem to reflect a binary distinction between 3D and 2D processing.

Since these distinct iTBRs were evident at posterior sensors, they might still relate to visual and attentional processing (see e.g., Li et al., 2020; Malik et al., 2015). While Tang et al. (2022) provided evidence for the differentiation of virtual 2D and 3D materials by the TBR until 300 ms after stimulus onset, our results shed light on subsequent time windows up to 750 ms after stimulus onset. Since the iTBR is associated with top‐down cognitive control, in particular mirroring the coordination of flexible, rapidly working networks (Cohen & Donner, 2013), the posterior iTBR might mirror higher stages of visual processing. For example, the posterior iTBR might be related to the maintenance of an object representation or an advancing manifestation of this representation based on visual input. More subtle differences in the objects beyond three‐dimensionality might be decisive at this stage, allowing for increasing differentiation between the conditions over time, whereas immediately after onset it only revealed differences between 2D and virtual 3D. However, the iTBR has predominantly been examined at midcentral to midfrontal sensors, while posterior regional means are only sparsely included in analyses (for exceptions see Bossi et al., 2020; Kisker et al., 2024), which impedes the functional interpretation of the differences found and requires further research. However, since the pattern of results is different from that of the midfrontal cluster, it can be assumed that the two regional peaks reflect functionally different processes that need further examination.

## 5 LIMITATIONS

The study was designed to realize a triangular comparison between planar 2D, virtual 3D and real‐world 3D objects. Visual features of the presented objects, like size, resulting viewing angle and texture were matched between conditions. Moreover, the generally possible interactions were matched (i.e., bringing the object closer, rotating it around all axes). We found this match to be crucial since a previous study revealed that the neural response is modulated by the perceived graspability of presented objects: When comparing real‐world objects and pictures of these objects, the difference in the neural response was smaller if the real‐world objects were not physically graspable due to a barrier (Fairchild et al., 2021).

However, the haptic sensation during the interaction with the objects was not matched. The RL condition provided a highly congruent match between the visible object, its texture and its contour. In the PC condition, participants experienced the same haptics for all objects with the computer mouse offering an incongruent, unchanging sensation. In contrast, the VR condition provided no haptic sensation at all, albeit hand tracking allowed for realistic interactions in terms of natural hand movements. Consequently, we cannot rule out that the congruence of the haptic sensation has an effect on our results. Yet the analyses are based on the mere presentation of the original objects. Albeit motor planning might already take place at this stage (e.g., Fairchild et al., 2021), all objects were generally interactable, while haptic sensations occurred only after the time window of interest.

Moreover, the topographical distribution of the iTBR indicated an orbitofrontal component reaching a strong negative amplitude in the VR condition, and a slightly less negative amplitude in the RL condition. This component was carefully counterchecked by an examination of the ICA and the cortical sources of the reported comparisons. The centre of gravities for these comparisons were located in cortical regions, not the eyeballs. Albeit the influence of the eye muscles cannot be completely ruled out, the degrees of freedom for eye movements are higher in the RL condition. Consequently, if the abovementioned orbitofrontal component was primarily driven by eye movements, we would have assumed a stronger amplitude in the RL condition compared to the VR condition, which is why, in combination with the counterchecks, we consider it unlikely that the frontal negative amplitude distribution is due to differences in eye movements.

## 6 CONCLUSIONS

In summary, our results demonstrate the potential to investigate visuospatial processing of real‐world objects under controlled laboratory conditions by means of a triangular comparison. Specifically, we demonstrate that the transferability of results obtained from planar 2D and virtual 3D conditions to real‐world conditions depends on whether primarily sensory stimulus features, as reflected in the evoked theta‐band response (eTBR), or more complex cognitive processes, as reflected in the induced theta‐band response (iTBR), are of interest.

Our results indicate that the eTBR does not binarily differentiate between the visual perception of 2D and 3D stimuli but between virtual 3D and real‐world 3D stimuli as well. However, the differences found were of quantitative rather than qualitative nature, indicating that the same process might be at work, with the VR condition intermediate between the planar PC condition and the RL condition.

Yet most importantly, the cognitive load during visuospatial processing as mirrored in the iTBR differed between planar 2D objects and both, virtual 3D and real‐world 3D objects. Yet only moderate evidence was found for the equivalence of the latter two. Against this background, they are not to be equated but would lead to the same theoretical conclusions about the underlying processes, which lowers the affordances to investigate these higher cognitive processes under realistic but controlled conditions. In contrast, the posterior iTBR seems to play another functional role in visual information processing than both the midfrontal iTBR and the posterior eTBR, which remains to be further examined.

## AUTHOR CONTRIBUTIONS

**Joanna Kisker:** Conceptualization (equal); methodology (equal); software (equal); investigation (equal); formal analysis (lead); visualization (lead); writing—original draft (lead); writing—review and editing (lead). **Marike Johnsdorf:** Conceptualization (equal); methodology (equal); software (equal); investigation (equal); formal analysis (supporting); visualization (supporting); writing—review and editing (supporting). **Merle Sagehorn:** Conceptualization (equal); investigation (supporting); writing—review and editing (supporting). **Thomas Hofmann:** Conceptualization (equal); resources (equal); writing—review and editing (supporting). **Thomas Gruber:** Conceptualization (equal); methodology (equal); funding acquisition (equal); supervision (equal); project administration (equal); resources (equal); writing—review and editing (supporting). **Benjamin Schöne:** Conceptualization (equal); methodology (equal); funding acquisition (equal); supervision (equal); project administration (equal); writing—review and editing (supporting).

## CONFLICT OF INTEREST STATEMENT

The authors declare that the research was conducted in the absence of any commercial or financial relationships that could be construed as a potential conflict of interest.

## ETHICS STATEMENT

The studies involving human participants were conducted in accordance with the declaration of Helsinki, as well as reviewed and approved by the local ethic committee of Osnabrück University, Germany (reference: Ethik 5/2023). The participants provided their written informed consent to participate in this study.

### PEER REVIEW

The peer review history for this article is available at https://www.webofscience.com/api/gateway/wos/peer-review/10.1111/ejn.16634.

## Supporting information

S1‐S5. Supplementary tables of mixed ANOVAs including the additional factor “PRESENTATION” to determine whether solely the response to first presentations or the mean value from the responses to first and second presentations should be analysed (see methods, statistical analyses).

## Data Availability

The datasets presented in this study can be found in online repositories. The names of the repository and accession number(s) can be found at OSF. All data covered in this publication is located in a subfolder entitled “*Visual information processing of 2D and virtual 3D and real 3D objects as marked by theta band responses*” of the following OSF directory: https://osf.io/6trmu/?view_only=6229545683e540609783fcc3ad862a0a. Exemplary video material of the three encoding modalities can be found online in the subfolder “*Exemplary video recordings of the encoding modalities*” at OSF (https://osf.io/6trmu/?view_only=6229545683e540609783fcc3ad862a0a).
