# Supplementary material for: Visual information processing of 2D, virtual 3D and real‐world objects marked by theta band responses: Visuospatial processing and cognitive load as a function of modality
Source: Eur J Neurosci. 2024 Dec 9;61(1):e16634. doi: 10.1111/ejn.16634 (PMC11664642; doi:10.1111/ejn.16634)
Supplement: Supplementary file 1 — S1‐S5. Supplementary tables of mixed ANOVAs including the additional factor “PRESENTATION” to determine whether solely the response to first presentations or the mean value from the responses to first and second presentations should be analysed (see methods, statistical analyses). [file EJN-61-0-s001.pdf]

Supplementary Material

Since the differentiation of the first presentation and the second presentation of each object was not of interest for the research objective at hand, a preceding mixed ANOVA was applied to check for any main effects or interactions of the within-factor presentation.
In case the ANOVA indicated a main effect of, or interaction with the factor presentation, only the responses to first presentation were further analyzed to prevent the influence of possible habituation or sensitization. Otherwise, the data were averaged across first and second presentations for further analyses. The main effects and interactions leading to the decision to analyze the response to first presentations only are highlighted in light grey in this document.

***S1.* 2 x 2 x 3 mixed ANOVA for the evoked theta band response (eTBR) for the central electrode cluster including the within-factors PRESENTATION (first, second) and TIMING (0-300ms, 300-600ms), and the between-factor GROUP (PC, VR, RL).**

Tests of Within-Subjects Effects

Source		Type III Sum of Squares	df	Mean Square	F	Sig.	Partial η^2
presentation	Sphericity assumed	1360.486	1	1360.486	117.118	<.001	.550
	Greenhouse-Geisser	1360.486	1.000	1360.486	117.118	<.001	.550
	Huynh-Feldt (HF)	1360.486	1.000	1360.486	117.118	<.001	.550
	Lower Bound	1360.486	1.000	1360.486	117.118	<.001	.550
presentation * group	Sphericity assumed	182.693	2	91.346	7.864	<.001	.141
	Greenhouse-Geisser	182.693	2.000	91.346	7.864	<.001	.141
	Huynh-Feldt (HF)	182.693	2.000	91.346	7.864	<.001	.141
	Lower Bound	182.693	2.000	91.346	7.864	<.001	.141
error(presentation)	Sphericity assumed	1115.169	96	11.616			
	Greenhouse-Geisser	1115.169	96.000	11.616			
	Huynh-Feldt (HF)	1115.169	96.000	11.616			
	Lower Bound	1115.169	96.000	11.616			

timing	Sphericity assumed	70.131	1	70.131	2.970	.088	.030
	Greenhouse-Geisser	70.131	1.000	70.131	2.970	.088	.030
	Huynh-Feldt (HF)	70.131	1.000	70.131	2.970	.088	.030
	Lower Bound	70.131	1.000	70.131	2.970	.088	.030
timing * group	Sphericity assumed	17.581	2	8.791	.372	.690	.008
	Greenhouse-Geisser	17.581	2.000	8.791	.372	.690	.008
	Huynh-Feldt (HF)	17.581	2.000	8.791	.372	.690	.008
	Lower Bound	17.581	2.000	8.791	.372	.690	.008
error(timing)	Sphericity assumed	2266.810	96	23.613			
	Greenhouse-Geisser	2266.810	96.000	23.613			
	Huynh-Feldt (HF)	2266.810	96.000	23.613			
	Lower Bound	2266.810	96.000	23.613			
presentation * timing	Sphericity assumed	2.767	1	2.767	.809	.371	.008
	Greenhouse-Geisser	2.767	1.000	2.767	.809	.371	.008
	Huynh-Feldt (HF)	2.767	1.000	2.767	.809	.371	.008
	Lower Bound	2.767	1.000	2.767	.809	.371	.008
presentation * timing * group	Sphericity assumed	3.165	2	1.582	.462	.631	.010
	Greenhouse-Geisser	3.165	2.000	1.582	.462	.631	.010
	Huynh-Feldt (HF)	3.165	2.000	1.582	.462	.631	.010
	Lower Bound	3.165	2.000	1.582	.462	.631	.010
error(presentation *timing)	Sphericity assumed	328.480	96	3.422			
	Greenhouse-Geisser	328.480	96.000	3.422			
	Huynh-Feldt (HF)	328.480	96.000	3.422			

	Lower Bound	328.480	96.000	3.422			

Test of Between-Subjects Effects						
Source	Type III Sum of Squares	df	Mean Square	F	Sig.	Partial η^2
Intercept	30932.583	1	30932.583	339.699	<.001	.780
group	105.145	2	52.573	.577	.563	.012
Error	8741.647	96	91.059			

***S2.* 2 x 2 x 3 mixed ANOVA for the evoked theta band response (eTBR) for the parietal electrode cluster including the within-factors PRESENTATION (first, second) and TIMING (0-300ms, 300-600ms). and the between-factor GROUP (PC, VR, RL).**

Tests of Within-Subjects Effects							
Source		Type III Sum of Squares	df	Mean Square	F	Sig.	Partial η^2
presentation	Sphericity assumed	1279.530	1	1279.530	248.971	<.001	.722
	Greenhouse-Geisser	1279.530	1.000	1279.530	248.971	<.001	.722
	Huynh-Feldt (HF)	1279.530	1.000	1279.530	248.971	<.001	.722
	Lower Bound	1279.530	1.000	1279.530	248.971	<.001	.722
presentation * group	Sphericity assumed	295.766	2	147.883	28.775	<.001	.375
	Greenhouse-Geisser	295.766	2.000	147.883	28.775	<.001	.375
	Huynh-Feldt (HF)	295.766	2.000	147.883	28.775	<.001	.375
	Lower Bound	295.766	2.000	147.883	28.775	<.001	.375
Error(presentation)	Sphericity assumed	493.370	96	5.139			
	Greenhouse-Geisser	493.370	96.000	5.139			
	Huynh-Feldt (HF)	493.370	96.000	5.139			

	Lower Bound	493.370	96.000	5.139			
timing	Sphericity assumed	78.422	1	78.422	10.009	.002	.094
	Greenhouse-Geisser	78.422	1.000	78.422	10.009	.002	.094
	Huynh-Feldt (HF)	78.422	1.000	78.422	10.009	.002	.094
	Lower Bound	78.422	1.000	78.422	10.009	.002	.094
timing * group	Sphericity assumed	34.359	2	17.180	2.193	.117	.044
	Greenhouse-Geisser	34.359	2.000	17.180	2.193	.117	.044
	Huynh-Feldt (HF)	34.359	2.000	17.180	2.193	.117	.044
	Lower Bound	34.359	2.000	17.180	2.193	.117	.044
Error(timing)	Sphericity assumed	752.193	96	7.835			
	Greenhouse-Geisser	752.193	96.000	7.835			
	Huynh-Feldt (HF)	752.193	96.000	7.835			
	Lower Bound	752.193	96.000	7.835			
presentation * timing	Sphericity assumed	8.435	1	8.435	4.802	.031	.048
	Greenhouse-Geisser	8.435	1.000	8.435	4.802	.031	.048
	Huynh-Feldt (HF)	8.435	1.000	8.435	4.802	.031	.048
	Lower Bound	8.435	1.000	8.435	4.802	.031	.048
presentation * timing * group	Sphericity assumed	1.935	2	.967	.551	.578	.011
	Greenhouse-Geisser	1.935	2.000	.967	.551	.578	.011
	Huynh-Feldt (HF)	1.935	2.000	.967	.551	.578	.011
	Lower Bound	1.935	2.000	.967	.551	.578	.011
Error(presentation *timing)	Sphericity assumed	168.648	96	1.757			
	Greenhouse-Geisser	168.648	96.000	1.757			

	Huynh-Feldt (HF)	168.648	96.000	1.757			
	Lower Bound	168.648	96.000	1.757			

Test of Between-Subjects Effects

Source	Type III Sum of Squares	df	Mean Square	F	Sig.	Partial η²
Intercept	11007.481	1	11007.481	470.336	<.001	.830
group	90.482	2	45.241	1.933	.150	.039
Error	2246.728	96	23.403			

***S3.* 2 x 2 x 3 mixed ANOVA for the evoked theta band response (eTBR) for the posterior electrode cluster including the within-factors PRESENTATION (first, second) and TIMING (0-300ms, 300-600ms). and the between-factor GROUP (PC, VR, RL).**

Tests of Within-Subjects Effects

Source		Type III Sum of Squares	df	Mean Square	F	Sig.	Partial η²
presentation	Sphericity assumed	3930.461	1	3930.461	261.037	<.001	.731
	Greenhouse-Geisser	3930.461	1.000	3930.461	261.037	<.001	.731
	Huynh-Feldt (HF)	3930.461	1.000	3930.461	261.037	<.001	.731
	Lower Bound	3930.461	1.000	3930.461	261.037	<.001	.731
presentation * group	Sphericity assumed	93.290	2	46.645	3.098	.050	.061
	Greenhouse-Geisser	93.290	2.000	46.645	3.098	.050	.061
	Huynh-Feldt (HF)	93.290	2.000	46.645	3.098	.050	.061
	Lower Bound	93.290	2.000	46.645	3.098	.050	.061
Error(presentation)	Sphericity assumed	1445.482	96	15.057			
	Greenhouse-Geisser	1445.482	96.000	15.057			

	Huynh-Feldt (HF)	1445.482	96.000	15.057			
	Lower Bound	1445.482	96.000	15.057			
timing	Sphericity assumed	95.659	1	95.659	4.092	.046	.041
	Greenhouse-Geisser	95.659	1.000	95.659	4.092	.046	.041
	Huynh-Feldt (HF)	95.659	1.000	95.659	4.092	.046	.041
	Lower Bound	95.659	1.000	95.659	4.092	.046	.041
timing * group	Sphericity assumed	2.216	2	1.108	.047	.954	.001
	Greenhouse-Geisser	2.216	2.000	1.108	.047	.954	.001
	Huynh-Feldt (HF)	2.216	2.000	1.108	.047	.954	.001
	Lower Bound	2.216	2.000	1.108	.047	.954	.001
Error(timing)	Sphericity assumed	2244.254	96	23.378			
	Greenhouse-Geisser	2244.254	96.000	23.378			
	Huynh-Feldt (HF)	2244.254	96.000	23.378			
	Lower Bound	2244.254	96.000	23.378			
presentation * timing	Sphericity assumed	54.565	1	54.565	11.901	<.001	.110
	Greenhouse-Geisser	54.565	1.000	54.565	11.901	<.001	.110
	Huynh-Feldt (HF)	54.565	1.000	54.565	11.901	<.001	.110
	Lower Bound	54.565	1.000	54.565	11.901	<.001	.110
presentation * timing * group	Sphericity assumed	6.731	2	3.366	.734	.483	.015
	Greenhouse-Geisser	6.731	2.000	3.366	.734	.483	.015
	Huynh-Feldt (HF)	6.731	2.000	3.366	.734	.483	.015

	Lower Bound	6.731	2.000	3.366	.734	.483	.015
Error(presentation *timing)	Sphericity assumed	440.133	96	4.585			
	Greenhouse-Geisser	440.133	96.000	4.585			
	Huynh-Feldt (HF)	440.133	96.000	4.585			
	Lower Bound	440.133	96.000	4.585			

Test of Between-Subjects Effects

Source	Type III Sum of Squares	df	Mean Square	F	Sig.	Partial η^2
Intercept	23241.422	1	23241.422	258.511	<.001	.729
group	1284.065	2	642.032	7.141	.001	.130
Error	8630.892	96	89.905			

***S4.* 2 x 2 x 3 mixed ANOVA for the induced theta band response (iTBR) for the midfrontal electrode cluster including the within-factors PRESENTATION (first, second) and TIMING (0-250ms, 250-500ms, 500-750ms), and the between-factor GROUP (PC, VR, RL).**

Tests of Within-Subjects Effects

Source		Type III Sum of Squares	df	Mean Square	F	Sig.	Partial η^2
presentation	Sphericity assumed	2107.755	1	2107.755	88.950	<.001	.481
	Greenhouse-Geisser	2107.755	1.000	2107.755	88.950	<.001	.481
	Huynh-Feldt (HF)	2107.755	1.000	2107.755	88.950	<.001	.481
	Lower Bound	2107.755	1.000	2107.755	88.950	<.001	.481
presentation * group	Sphericity assumed	27.276	2	13.638	.576	.564	.012
	Greenhouse-Geisser	27.276	2.000	13.638	.576	.564	.012
	Huynh-Feldt (HF)	27.276	2.000	13.638	.576	.564	.012

	Lower Bound	27.276	2.000	13.638	.576	.564	.012
Error(presentation)	Sphericity assumed	2274.801	96	23.696			
	Greenhouse-Geisser	2274.801	96.000	23.696			
	Huynh-Feldt (HF)	2274.801	96.000	23.696			
	Lower Bound	2274.801	96.000	23.696			
timing	Sphericity assumed	665.770	2	332.885	79.554	<.001	.453
	Greenhouse-Geisser	665.770	1.958	339.995	79.554	<.001	.453
	Huynh-Feldt (HF)	665.770	2.000	332.885	79.554	<.001	.453
	Lower Bound	665.770	1.000	665.770	79.554	<.001	.453
timing * group	Sphericity assumed	5.041	4	1.260	.301	.877	.006
	Greenhouse-Geisser	5.041	3.916	1.287	.301	.873	.006
	Huynh-Feldt (HF)	5.041	4.000	1.260	.301	.877	.006
	Lower Bound	5.041	2.000	2.521	.301	.741	.006
Error(timing)	Sphericity assumed	803.406	192	4.184			
	Greenhouse-Geisser	803.406	187.985	4.274			
	Huynh-Feldt (HF)	803.406	192.000	4.184			
	Lower Bound	803.406	96.000	8.369			
presentation * timing	Sphericity assumed	227.994	2	113.997	3.725	.026	.037
	Greenhouse-Geisser	227.994	1.093	208.556	3.725	.053	.037
	Huynh-Feldt (HF)	227.994	1.119	203.697	3.725	.052	.037
	Lower Bound	227.994	1.000	227.994	3.725	.057	.037

presentation * timing * group	Sphericity assumed	29.736	4	7.434	.243	.914	.005
	Greenhouse-Geisser	29.736	2.186	13.600	.243	.804	.005
	Huynh-Feldt (HF)	29.736	2.239	13.283	.243	.809	.005
	Lower Bound	29.736	2.000	14.868	.243	.785	.005
Error(presentation *timing)	Sphericity assumed	5876.453	192	30.607			
	Greenhouse-Geisser	5876.453	104.948	55.994			
	Huynh-Feldt (HF)	5876.453	107.451	54.690			
	Lower Bound	5876.453	96.000	61.213			

Test of Between-Subjects Effects

Source	Type III Sum of Squares	df	Mean Square	F	Sig.	Partial η^2
Intercept	386.460	1	386.460	2.239	.138	.023
group	1578.371	2	789.185	4.572	.013	.087
Error	16571.786	96	172.623			

***S5.* 2 x 2 x 3 Mixed ANOVA for the induced theta band response (iTBR) for the posterior electrode cluster including the within-factors PRESENTATION (first, second) and TIMING (0-250ms, 250-500ms, 500-750ms), and the between-factor GROUP (PC, VR, RL).**

Tests of Within-Subjects Effects

Source		Type III Sum of Squares	df	Mean Square	F	Sig.	Partial η^2
presentation	Sphericity assumed	1374.746	1	1374.746	37.928	<.001	.283
	Greenhouse-Geisser	1374.746	1.000	1374.746	37.928	<.001	.283
	Huynh-Feldt (HF)	1374.746	1.000	1374.746	37.928	<.001	.283
	Lower Bound	1374.746	1.000	1374.746	37.928	<.001	.283

presentation * group	Sphericity assumed	234.692	2	117.346	3.237	.044	.063
	Greenhouse-Geisser	234.692	2.000	117.346	3.237	.044	.063
	Huynh-Feldt (HF)	234.692	2.000	117.346	3.237	.044	.063
	Lower Bound	234.692	2.000	117.346	3.237	.044	.063
Error(presentation)	Sphericity assumed	3479.607	96	36.246			
	Greenhouse-Geisser	3479.607	96.000	36.246			
	Huynh-Feldt (HF)	3479.607	96.000	36.246			
	Lower Bound	3479.607	96.000	36.246			
timing	Sphericity assumed	925.353	2	462.677	61.331	<.001	.390
	Greenhouse-Geisser	925.353	1.802	513.409	61.331	<.001	.390
	Huynh-Feldt (HF)	925.353	1.873	494.042	61.331	<.001	.390
	Lower Bound	925.353	1.000	925.353	61.331	<.001	.390
timing * group	Sphericity assumed	116.760	4	29.190	3.869	.005	.075
	Greenhouse-Geisser	116.760	3.605	32.391	3.869	.007	.075
	Huynh-Feldt (HF)	116.760	3.746	31.169	3.869	.006	.075
	Lower Bound	116.760	2.000	58.380	3.869	.024	.075
Error(timing)	Sphericity assumed	1448.424	192	7.544			
	Greenhouse-Geisser	1448.424	173.028	8.371			
	Huynh-Feldt (HF)	1448.424	179.811	8.055			
	Lower Bound	1448.424	96.000	15.088			
presentation * timing	Sphericity assumed	375.208	2	187.604	6.767	.001	.066

	Greenhouse-Geisser	375.208	1.196	313.650	6.767	.007	.066
	Huynh-Feldt (HF)	375.208	1.228	305.515	6.767	.007	.066
	Lower Bound	375.208	1.000	375.208	6.767	.011	.066
presentation * timing * group	Sphericity assumed	210.714	4	52.678	1.900	.112	.038
	Greenhouse-Geisser	210.714	2.393	88.072	1.900	.146	.038
	Huynh-Feldt (HF)	210.714	2.456	85.787	1.900	.144	.038
	Lower Bound	210.714	2.000	105.357	1.900	.155	.038
Error(presentation *timing)	Sphericity assumed	5322.853	192	27.723			
	Greenhouse-Geisser	5322.853	114.841	46.350			
	Huynh-Feldt (HF)	5322.853	117.899	45.148			
	Lower Bound	5322.853	96.000	55.446			

Test of Between-Subjects Effects

Source	Type III Sum of Squares	df	Mean Square	F	Sig.	Partial η^2
Intercept	4778.815	1	4778.815	20.861	<.001	.179
group	1409.004	2	704.502	3.075	.051	.060
Error	21991.237	96	229.075			
